# Effectiveness of Physiotherapy Exercises on Pain, Range of Motion, and Quality of Life in Patients With Ankylosing Spondylitis: A Case Report

**DOI:** 10.7759/cureus.53338

**Published:** 2024-01-31

**Authors:** Vishal U Telrandhe, Swapna Jawade, Rutuja Nimbalkar

**Affiliations:** 1 Department of Musculoskeletal Physiotherapy, Ravi Nair Physiotherapy College, Datta Meghe Institute of Higher Education and Research, Wardha, IND

**Keywords:** case report, rehabilitation, pilates exercise, mckenzie exercise, physical therapy, ankylosing spondylitis

## Abstract

Ankylosing spondylitis (AS) is an inflammatory disease that affects the entire body. Immune cells positive for human leukocyte antigen B27 are thought to be involved in the pathophysiology. AS is caused by inflammation of the spinal joints, leading to stiffness and reduced spinal movement. This case study describes a 38-year-old man who suffered from hip problems and back pain that worsened over eight years. This study investigates how well physiotherapy exercises can help patients with AS manage their pain, increase their range of motion, and improve their overall quality of life. It examines the effects of a structured physiotherapy intervention on pain levels, functional mobility, and general well-being in a particular group of patients. Quantitative measures are used in the assessment to assess changes in pain intensity, range of motion, and quality of life. These measures offer important new information about the potential advantages of physiotherapy as a supplemental treatment for AS. The results add to the increasing amount of data demonstrating the role of physiotherapy exercises as an additional therapeutic approach for people with AS.

## Introduction

The axial skeleton is the main part of the body affected by ankylosing spondylitis (AS), a rheumatic condition with chronic inflammation that causes stiffness and back discomfort. As the disease progresses, the costovertebral joints become implicated, increasing thoracic kyphosis with limited chest excursion, slanting of the lumbar spine, and bony ankyloses [1]. About 0.25% of the people in India are thought to be afflicted by AS, with an incidence of 7 per 10,000 people [2,3]. Early diagnosis is essential for effective treatment of the condition. However, in addition to being difficult, after seven to ten years of symptom onset, the diagnosis of AS is frequently ignored or delayed [4]. Overall, anti-inflammatory drugs, physical activity, and surgery, if necessary, have been used to treat AS. In the clinical practice list of suggestions for managing AS, exercise is strongly advised [5,6]. The goals of AS-specific activities have traditionally concentrated on axial and peripheral mobility of joint tasks, strengthening of antigravity muscles, and stretching of tight muscles with cardiopulmonary endurance training to improve or maintain physical function and posture. However, there is little research on the long-term advantages of workouts designed specifically for AS [7]. The goal of treating AS is to relieve symptoms, minimize functional limits, maintain spinal flexibility, and promote normal posture [8]. As a result, physiotherapy is extremely crucial in the treatment of AS. A physiotherapist-supervised patient training and exercise program may help patient’s symptoms and teach them how to manage AS effectively and independently for the rest of their lives, minimizing the financial impact on the healthcare system [9]. For treating AS, relevant clinical practice guidelines make exercise a top recommendation [2,10]. However, there is little proof of the long-term advantages of AS-specific workouts [7].

## Case presentation

Patient information

A 38-year-old man complained of lower back pain that worsened over time and limited mobility of his hip joint and spine, making it difficult for him to stand upright and perform everyday tasks for five years. He had similar episodes 10 years ago when he started experiencing pain in his lower back and hip joint, for which he had undergone medical treatment. He was diagnosed with tuberculosis of the hip joint and was hospitalized and treated with medication. After four months, the patient discontinued these medications because the symptoms were reduced, and after five years, the patient’s symptoms increased in the form of pain and restrictions in the hip joint and spine. The patient was subsequently treated for tuberculosis in the hip. However, the symptoms returned, waking him up at night and causing him to feel stiff in the morning. He had no history of falls, diabetes, or hypertension. Table 1 shows the timeline of events.

**Table 1:** Timeline of events.

Events	Date
Date of first experiencing symptoms	20/09/2013
Date of quitting medication	23/01/2014
Date of the second time experiencing the same symptoms	30/04/2019
Date of recently experienced symptoms	19/05/2023
Date of recent hospitalization	04/09/2023
Date of discharge	18/11/2023

Clinical findings

Musculoskeletal examination revealed kyphosis, a forward bowed neck, and flexion deformities in both hip joints. Lateral and anterior flexion of the lumbar spine was lost. The modified results of the Schober test were favorable. With limited mobility of the hip joint, Patrick’s test reproduced pain throughout the lumbosacral region on both sides. His chest extent was only 1 cm above or below the nipple, armpit, and sternum. According to the Numerical Pain Rating Scale, the patient complained of a pain score of 7/10. The pain was dull aching, with a rapid onset and slow progression. Movement, prolonged standing, and constant standing aggravated the patient’s symptoms. Rest and medication helped improve the symptoms. Grade 2 tenderness was visible in the lumbar spine, i.e., the patient was in pain and winced when palpated. As the patient could not bend, the spinal areas were inaccessible. According to resistance isometry, the patient’s strength for the flexors of the spine was weak and painful, while it was also weak and painful for the lateral flexors of the spine. Table 2 shows the range of motion before the procedure.

**Table 2:** Pre-intervention lumbar range of motion.

Movement	Patient’s range of motion	Normal range of motion
Lumbar side flexion on both sides	0°–10°	0°–35°
Lumbar flexion	0°–25°	0°–80°
Lumbar extension	0°–10°	0°–25°

Muscle strength was assessed according to the Oxford scale. Table 3 shows the findings of pre-intervention manual muscle testing.

**Table 3:** Pre-intervention manual muscle testing according to Oxford grading. Fair (3-): some but not complete range of motion against gravity. Normal (5+): complete range of motion against gravity with maximal resistance.

Movement	Strength
Lumbar side flexion	Fair-
Lumbar flexion	Fair-
Lumbar extension	Fair-

Diagnostic assessment

**Figure 1:**
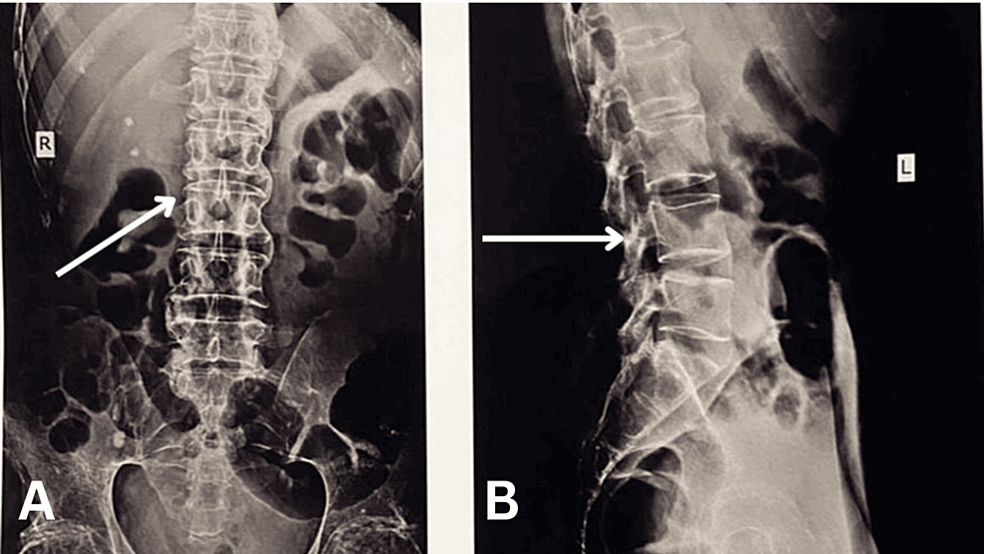
Lumbosacral spine X-ray. (A) Anteroposterior view showing bamboo spine appearance. (B) Lateral view of the lumbosacral spine where curvature has disappeared.

Figure 1 shows the radiographs of the lumbosacral spine, which verified the probable diagnosis of AS.

Physiotherapy treatment

Table 4 presents the detailed physiotherapy intervention given to the patient in two phases for four weeks.

**Table 4:** Summary of therapeutic intervention. ROM: range of motion

Rehabilitation phases	Goals	Dosage and interventions
Phase 1: Early phase (day 1 one week 2)	Patient education	Patient education in physiotherapy is a crucial part of the treatment process because it gives patients the knowledge and confidence to understand their condition, take an active role in their rehabilitation, and take charge of their own health to prevent re-injury and enhance daily activities
	To improve ROM	Gentle stretching exercises such as neck stretch, shoulder stretch, hip flexor stretch, and spinal can be given for 15–30 seconds for one side, and then the same for the other side. These exercises should be done gently and within a pain-free range of motion to prevent any potential strain or injury
	To improve cardiorespiratory conditioning	Deep breathing exercises should start with 5–10 minutes and gradually increase as you become more comfortable and aim for around 10–15 breaths per minute. Thoracic expansion exercises for 5–10 minutes initially to preserve chest expansion and respiratory function, which can be impacted by AS-related chest involvement. Additionally, the chest muscles can be relaxed, and general mobility can be enhanced with these exercises
Phase 2: Intermediate phase (weeks 2–4)	To improve muscle strength	Strengthening exercises that can be beneficial for patients in the preoperative intermediate phase are core strengthening exercises, and static abdominal exercises (Position: Lie on your back with knees bent and feet flat on the floor. Procedure: (1) inhale deeply, expanding your abdomen; (2) exhale slowly while pulling your navel toward your spine, engaging the abdominal muscles; (3) hold the contraction for 5–10 seconds, focusing on maintaining steady breathing. Repetitions: 8-10 repetitions), hip abductor and adductor strengthening exercises, dynamic quadriceps, and combined strengthening exercises with gentle stretching routines to maintain flexibility and muscle tightness in areas surrounding the spine, hips, and shoulders. McKenzie extension exercises (initially, 5–10 repetitions per session) to increase the mobility of the spine and strengthen paraspinal muscles. Pilates to improve patients’ quality of life and mobility of the spine. Static cycling to improve and maintain the strength of the lower limb (10 repetitions × 2 sets)
	Joint mobility	Incorporate exercises that involve multiple joints, such as arm circles, leg swings, and torso twists, to improve overall flexibility and range of motion throughout the body. It is important to perform these joint mobility exercises regularly but gently, staying within a pain-free range of motion to prevent any potential discomfort or injury

Follow-up and outcomes measures

Ranges were assessable to some extent. Table 5 shows the outcome measures, and Table 6 shows the post-intervention range of motion.

**Table 5:** Outcome measures.

Outcome measures	Pre-intervention	Post-intervention
Numerical Pain Rating Scale score	7/10	4/10
Modified Oswestry Disability score	29/50	24/50

**Table 6:** Post-intervention range of motion.

Movement	Patient’s range of motion	Normal range of motion
Lumbar side flexion	0°–25°	0°–35°
Lumbar flexion	0°–50°	0°–80°
Lumbar extension	0°–15°	0°–25°

Post-intervention muscle strength was assessed and the findings are presented in Table 7.

**Table 7:** Post-intervention manual muscle testing according to Oxford grading. Fair (3+): complete range of motion against gravity with minimal resistance. Normal (5+): complete range of motion against gravity with maximum resistance.

Movement	Strength
Lumbar side flexion	Fair+
Lumbar flexion	Fair+
Lumbar extension	Fair+

## Discussion

There is a broad understanding that the ankylosis of the rib cage, frequently present in advanced AS, restricts chest expansion and vital capacity [11]. Additionally, patients experience a significant reduction in their ability to exert themselves due to the atrophic nature of the intercostal muscles, which are frequently used for breathing [12]. Pilates and McKenzie’s approaches focus on correcting vertebral statics through movements that maintain both spine mobility and body alignment, maintaining pulmonary ventilation, and preventing deterioration [13]. Additionally, maintaining respiratory compliance for persons with AS requires incorporating breathing exercises with exercises aimed at achieving or maintaining the standard spinal posture. According to the general consensus, these kinetic programs maintain the spine’s range of motion; neutralize the pelvic muscles; relax the neck, scapula-humeral, and coxofemoral muscles; regulate breathing with controlled movements; and retrain the diaphragm.

Additionally, our findings supported earlier research about the effectiveness of non-pharmacological therapies, including physical activity, instruction, and physiotherapy, in lowering the symptoms of AS [14]. Regular exercise improves many outcomes, including functional and spinal mobility, even though the effects on disease activity are relatively minor. This is particularly true when compared to no intervention. It was previously recommended that the best disease management strategy is a multimodal strategy under the supervision of a physiotherapist followed by a home-based regimen [15]. Over nine years, the condition quickly worsened, with neck stiffness getting worse before back stiffness, chest excursions getting smaller, and spinal mobility getting more limited. Despite the typical AS symptoms, it took ten years to diagnose the condition and begin rehabilitation, primarily consisting of AS-specific exercises [12]. Long-term effects on this patient’s pain, stiffness, mobility, and function were observed after a six-day, 270-360-minute per week, supervised exercise program [16]. The goal of patient-specific physical therapy exercise interventions was to reduce or enhance musculoskeletal side effects such as pain, postural deformities, decreased mobility, and muscle strength, as well as secondary side effects such as changes in cardiorespiratory function, balance, and physical activity [2,10].

## Conclusions

This case study highlights the significant effectiveness of physical therapy exercises in addressing the various issues related to AS. The structured physiotherapy intervention demonstrated significant improvements in range of motion, a significant decrease in pain, and an overall improvement in the quality of life for individuals with AS. These results highlight the potential of physiotherapy as an instrumental and complementary therapeutic approach, and they are consistent with the increasing body of evidence that supports the inclusion of physiotherapy in the comprehensive management of AS. The nuanced insights of this study highlight the importance of individualized physiotherapeutic interventions in improving the quality of life for individuals with AS, in addition to providing valuable data to the body of existing literature. In the context of AS, physiotherapy exercises appear to be a promising means of improving patient outcomes and promoting a more comprehensive approach to care as we navigate the terrain of chronic inflammatory conditions.
